# Does Feeling Bad Lead to Eating Bad? How Emotion, Deliciousness (Mis)match, and Weight Status Alter Food Choice and ERPs

**DOI:** 10.3390/bs16081265

**Published:** 2026-07-23

**Authors:** Shen Mao, Jiali Sun, Yazhi Pang, Yuanluo Jing, Hong Yuan, Jia Zhao, Yong Liu

**Affiliations:** 1 Faculty of Psychology, Southwest University, Chongqing 400715, China; maoshen@email.swu.edu.cn (S.M.); sjl1455620654@email.swu.edu.cn (J.S.); xiaoshi.pang.20@alumni.ucl.ac.uk (Y.P.); yuanluo.jing.23@alumni.ucl.ac.uk (Y.J.); yuanyh@swu.edu.cn (H.Y.); jiazhao@swu.edu.cn (J.Z.); 2 Key Laboratory of Cognition and Personality, Ministry of Education, Southwest University, Chongqing 400715, China

**Keywords:** overweight individuals, food deliciousness, emotional eating, food decision-making, event-related potentials

## Abstract

Negative emotion and perceived food deliciousness are important factors contributing to unhealthy food choices, but their interaction during food decision-making remains unclear. This study examined how negative emotion and deliciousness congruency influence high-calorie food choices and related event-related potential (ERP) responses in overweight individuals (OWs) and normal-weight individuals (NWs). Fifty female college students, including 24 OWs and 26 NWs, completed a binary food choice task before and after negative and neutral emotion induction. Participants chose high-calorie foods more frequently and responded faster in the deliciousness-incongruent condition than in the deliciousness-congruent condition. Negative emotion increased high-calorie food choices among OWs but not among NWs. The deliciousness-incongruent condition elicited less negative N1 amplitudes and marginally less negative N2 amplitudes. P3 amplitudes were modulated by emotion, group, and deliciousness congruency, with negative emotion reducing P3 amplitudes in NWs but not in OWs. High-calorie food choices are jointly shaped by negative emotion, deliciousness congruency, and weight status. Deliciousness advantage facilitates high-calorie food choices, whereas negative emotion selectively increases such choices among OWs. ERP results suggest that these effects involve both early attentional processing and later evaluative processing.

## 1. Introduction

Overweight and obesity have become major global public health concerns. Epidemiological data indicate that more than one billion people worldwide were living with obesity in 2022 (Phelps et al., 2024). Moreover, overweight and obesity are closely associated with increased risks of cardiovascular disease, type 2 diabetes, several types of cancer, and premature mortality (Blüher, 2025). Unhealthy dietary patterns are among the key contributors to weight gain and the maintenance of obesity, particularly the excessive intake of foods high in calories, sugar, and fat (Rouhani et al., 2016). For individuals seeking to lose weight or control body weight, improving dietary habits and reducing unhealthy food choices are therefore of considerable importance. However, everyday food choices are influenced by multiple factors, including sociocultural contexts, food attributes, individual preferences, and situational factors, rather than being determined solely by health goals (Chen & Antonelli, 2020; Fernqvist et al., 2024). Accordingly, examining how individuals choose between different food options may help elucidate the psychological mechanisms underlying unhealthy eating behaviors associated with overweight and obesity.

According to the goal conflict model of eating, food choices often involve a goal conflict between enjoying palatable foods and controlling body weight (Stroebe et al., 2013; Stroebe, 2022). Palatable, high-calorie foods typically have high reward value and are more likely to capture attention, elicit food craving, and strengthen choice bias (Y. Liu et al., 2021; Bianco et al., 2023). Such food temptation may further activate hedonic eating goals while weakening the influence of weight control goals, thereby making individuals more susceptible to immediate palatable rewards. From a reward learning perspective, palatable high-calorie foods may acquire incentive value through repeated associations with pleasurable taste and prior eating experiences. Through learning processes, food cues associated with palatable high-calorie foods may evoke anticipatory reward responses and craving, thereby biasing attention and choice toward foods with higher hedonic value (van den Akker et al., 2018). In this process, food deliciousness often serves as a direct and powerful driving factor (Sullivan et al., 2015; Georgii et al., 2019). In the present study, perceived deliciousness refers to the subjective palatability of a food, as judged by independent raters based on its visual appearance and their prior eating experiences, rather than actual taste experience. Individuals commonly associate foods high in sugar, fat, or energy density with greater palatability and reward value. On the one hand, highly palatable foods often contain larger amounts of fat, sugar, sodium, or carbohydrates, which may enhance the rewarding effects of food intake and promote appetite and energy consumption (Jun et al., 2025; Fazzino et al., 2019). On the other hand, consumers often show an “unhealthy = tasty” intuition, namely the tendency to perceive unhealthy foods as tastier than healthy foods, and this cognitive tendency may increase preferences for high-calorie foods (Raghunathan et al., 2006; Paakki et al., 2022).

Although high-calorie foods are often perceived as more palatable, calorie content and perceived deliciousness are not necessarily equivalent. Calorie content is an objective nutritional property related to energy density and is often associated with health or weight control considerations, whereas perceived deliciousness reflects a subjective hedonic attribute related to expected taste, palatability, and reward value. Previous food choice studies have operationally distinguished energy content from perceived deliciousness by constructing high- and low-energy food pairs that were either matched or differed in perceived tastiness (Van Der Laan et al., 2014). Neuroimaging evidence further suggests that taste- and health-related attributes may be differentially integrated into subjective value signals during food choice (Hare et al., 2009). Together, these findings indicate that calorie content and deliciousness can be distinguished during food choice. Previous studies have examined calorie content, emotional eating, and food reward separately; however, relatively little is known about how deliciousness and calorie content interact during food choice under different emotional states, particularly among overweight individuals. Research on food decision-making suggests that calorie content and deliciousness may contribute differently to subjective value computation, and individuals’ final choices may depend on how these attributes are weighted and integrated during decision-making, particularly under different emotional states (Suzuki, 2022). On this basis, it is necessary to further examine how deliciousness interacts with calorie content under different emotional states, and whether this process differs between overweight and normal-weight individuals.

The goal conflict model of eating further proposes that when individuals are under high cognitive load, such as when experiencing intense negative emotions, hedonic eating goals are more likely to become dominant, thereby increasing the likelihood of overeating or choosing high-calorie foods. The emotion-imbued choice model suggests that current emotions may influence decision-making by shaping the evaluation of choice options, the dimensions to which decision makers attend, the depth of information processing, and the motivational goals activated during evaluation (Lerner et al., 2015). In food choice contexts, such affective influences may contribute to shifts in the relative salience of palatable food rewards and weight control goals. Consistent with this possibility, emotions may influence food choice, weaken cognitive control over eating, and promote eating as a means of regulating unpleasant affect (Macht, 2008). Under negative emotional states, individuals are more prone to overconsume energy-dense, highly palatable foods rich in sugar and fat, a tendency commonly referred to as emotional eating (Fuente González et al., 2022; Ha & Lim, 2023). From the perspective of emotion regulation, the rewarding properties of highly palatable foods may counteract negative emotions; thus, emotional eating strategies are more likely to emerge under negative emotional states (Reichenberger et al., 2020; Klatzkin et al., 2021). Although emotional eating may alleviate negative emotions in the short term, it represents a maladaptive emotion-regulation strategy in the long term, as it may lead to increased intake of high-calorie foods, weight gain, obesity maintenance, difficulty losing weight, and a renewed cycle of emotional eating (Dakanalis et al., 2023). Recent evidence also suggests that binge eating behavior is closely associated with depression severity and BMI, further supporting the link between negative affect, maladaptive eating behavior, and weight-related characteristics (Özbay et al., 2025).

This negative emotion-related tendency in food choice may be more pronounced among overweight individuals (OWs). Compared with normal-weight adults, adults with obesity show higher levels of emotional eating (Vasileiou & Abbott, 2023). When exposed to visual food cues, OWs exhibit stronger responses in brain regions associated with reward processing, whereas normal-weight individuals (NWs) show greater responses in regions related to inhibitory control (Meng et al., 2020). These findings suggest that heightened emotional eating tendencies and stronger food reward responses may jointly increase the likelihood that OWs are attracted to high-calorie, highly palatable foods under negative emotional states. Therefore, examining food choices among OWs across different emotional states may not only help reveal how negative emotion influences bias toward high-calorie foods, but also further clarify the mechanisms that trigger unhealthy eating behaviors associated with overweight and obesity.

Food decision-making involves multiple sequential processing stages, including visual attentional allocation, the evaluation of food attributes, the monitoring of goal conflict, and the integration of decision value. Event-related potentials (ERPs) can reveal the neural dynamics of different stages during food choice and have therefore been widely used in research on food-related cognition and dietary control (Carbine et al., 2018; Suzuki, 2022; Zsoldos et al., 2022). The N1 component is associated with early visual perceptual processing and attentional selection (Schindler et al., 2018). Meule et al. (2013) found that high-calorie foods elicited less negative N1 amplitudes than low-calorie foods, suggesting that food types can be differentiated at an early stage of processing. The N2 component is commonly associated with conflict monitoring and response inhibition (Folstein & Van Petten, 2008). Previous research has shown that high-calorie foods elicit less negative N2 amplitudes (Y. Liu et al., 2022). P3 is one of the late positive components frequently examined in food-related ERP studies and is generally considered to be associated with attentional resource allocation and decision-related processing (Polich, 2007; Carbine et al., 2018). In a food choice task, Kirsten et al. (2022) found that late ERP components such as the P3 were influenced by the caloric properties of foods and contextual factors. High-calorie or highly delicious food cues often elicit larger P3 amplitudes, which may reflect the allocation of greater processing resources to food reward cues (Biehl et al., 2020). In addition, the P3 is sensitive to emotional states and can reflect cognitive processes involved in food decision-making under negative emotion (Schnepper et al., 2020).

From the perspective of the goal conflict model of eating, food choices involving palatable high-calorie foods may reflect a conflict between hedonic eating goals and weight control goals (Stroebe et al., 2013; Stroebe, 2022). Previous food choice research has also treated choices between healthy or low-energy foods and more palatable high-energy foods as self-control dilemmas involving conflict monitoring (Van Der Laan et al., 2014). Therefore, calorie content and perceived deliciousness may have different implications for ERP responses because they are related to different choice-relevant goals. Specifically, N1 may reflect early attentional sensitivity to hedonic food cues, N2 may reflect conflict-related processing between hedonic eating and weight control goals, and P3 may reflect later evaluative processing and attentional resource allocation during food decision-making.

Taken together, food choice is influenced by calorie content, deliciousness, emotional state, and individual body weight. However, these factors have often been examined separately. Consequently, relatively little is known about how negative emotion and perceived deliciousness jointly influence high-calorie food choices, particularly among OWs. The goal conflict model of eating suggests that food choice depends on the relative dominance of hedonic eating goals and weight control goals. Negative emotion may increase the dominance of hedonic eating goals, whereas prior eating experiences may lead individuals to associate high-calorie foods with greater palatability, thereby allowing a deliciousness advantage to enhance the reward value of high-calorie foods. Weight status may further shape this process because OWs may differ from NWs in emotion-related eating tendencies and responses to food reward cues. Therefore, by jointly considering emotion, deliciousness congruency, and weight status, the present study examines under what conditions and for whom high-calorie food choices are most likely to occur, while also clarifying the temporal ERP correlates of this process.

Accordingly, the present study used an emotion induction paradigm and a binary food choice task (BFCT) to compare NWs and OWs under neutral and negative emotional states, focusing on the effects of deliciousness on food choice behavior and ERP responses. We hypothesized that, first, compared with the deliciousness-congruent condition, individuals would show a stronger preference for high-calorie foods in the deliciousness-incongruent condition, as reflected by a higher proportion of high-calorie choices and faster reaction times. Second, negative emotion would increase the bias toward high-calorie food choices among OWs, whereas this effect would be relatively weaker among NWs. Third, at the ERP level, the deliciousness-incongruent condition would elicit less negative N1 and N2 amplitudes. For the P3 component, given that high-calorie or highly delicious food cues often elicit larger P3 amplitudes and that P3 is sensitive to emotional states, we expected P3 amplitudes to be modulated by emotion, deliciousness congruency, and weight status.

## 2. Materials and Methods

### 2.1. Participants

A total of 61 female college students were recruited through social media advertisements on campus. After data collection, 11 participants who reported special dietary habits, such as dieting, were excluded. The final sample consisted of 50 participants, including 24 OWs and 26 NWs. Statistical power analysis was conducted using G*Power 3.1.9.7 (Heinrich Heine University Düsseldorf, Düsseldorf, Germany) for repeated-measures ANOVA assessing the within–between interaction. Assuming a medium effect size (*f* = 0.25), an α level of 0.05, two groups, and two measurement points, the total sample size of 50 yielded a statistical power of 0.934. Criteria for body mass index (BMI) grouping followed the World Health Organization (WHO) standards for Asian populations: NW (18.5 ≤ BMI < 25.0 kg/m^2^) and OW (BMI ≥ 25.0 kg/m^2^). Anthropometric measurements (height, weight, waist, and hip circumference) were obtained in the laboratory, and BMI and waist-to-hip ratio (WHR) were calculated based on these measurements. Written informed consent was secured from all participants, and the experimental protocol received approval from the Ethics Committee.

### 2.2. Self-Report Measures

#### 2.2.1. Positive and Negative Affect Schedule (PANAS)

PANAS was used to assess participants’ emotional states (Watson et al., 1988; Huang et al., 2003). It consists of two relatively independent subscales measuring positive affect (PA) and negative affect (NA). Participants rated their current affective experience, with higher scores indicating stronger affect. In this study, the PANAS was used to verify whether the emotion induction procedure successfully elicited the intended emotional states. In the present study, the Cronbach’s alpha coefficients for the PA and NA subscales were 0.897 and 0.846, respectively.

#### 2.2.2. Visual Analog Scale (VAS)

Individual differences in hunger, thirst, desire to eat, happiness, and sadness were assessed using the VAS (Flint et al., 2000). Each item was rated on a continuous line ranging from 0% to 100%, with 0% indicating “not at all” and 100% indicating “extremely strong.” Participants responded to five single-item questions: “How hungry do you feel right now?”, “How thirsty do you feel right now?”, “How strong is your current desire to eat?”, “How happy do you feel right now?”, and “How sad do you feel right now?” Hunger, thirst, and desire to eat were assessed to examine whether participants differed in subjective eating motivation across groups or time, whereas happiness and sadness were included as supplementary measures of emotional state in addition to PANAS.

### 2.3. Tools and Materials

#### 2.3.1. Emotional Stimulus Materials

Negative affective states were induced using a 4 min edited video depicting events related to the COVID-19 pandemic in China. The video consisted of several COVID-19-related clips from China and was used to elicit negative affective states. Previous validation (X. Liu et al., 2021) confirmed that this material primarily elicited negative affect, with standardized emotion ratings indicating relatively high levels of sadness (*M* = 71.03, *SD* = 19.43) and tension (*M* = 69.12, *SD* = 22.56). Neutral emotional states were induced using duration-matched landscape videos devoid of socially or emotionally salient content (Bian et al., 2021; Y. Liu et al., 2020; X. Liu et al., 2021). The efficacy of the emotion induction was verified using subjective affective scaling.

#### 2.3.2. Food Stimulus Materials

A standardized set of 100 food images was selected, including 50 high-calorie and 50 low-calorie food images. The food images were selected from the food image database (Li et al., 2022). Prior to the formal experiment, 200 participants with a similar cultural background to the formal participants rated the perceived deliciousness of the food images on a 9-point scale. Only images with mean deliciousness ratings above 4 were selected to reduce potential choice bias caused by strong dislike of particular foods. In addition, pleasure and familiarity were matched to ensure consistency across dimensions other than calorie content. Low-calorie foods had mean energy content ratings below 4, whereas high-calorie foods had mean energy content ratings above 6. All images were standardized for brightness, contrast, and visual complexity to ensure that low-level visual features were balanced across conditions. Each food pair consisted of one high-calorie food and one low-calorie food. Based on the deliciousness ratings of the two foods in each pair, food pairs were classified into two conditions: a congruent condition and an incongruent condition. In the congruent condition, the high-calorie and low-calorie foods were matched in deliciousness ratings, with 60 trials included. In the incongruent condition, the high-calorie foods were rated as more delicious than the low-calorie foods, including 60 trials with a 2-point rating difference and 60 trials with a 3-point rating difference.

#### 2.3.3. Binary Food Choice Task (BFCT)

**Figure 1:**
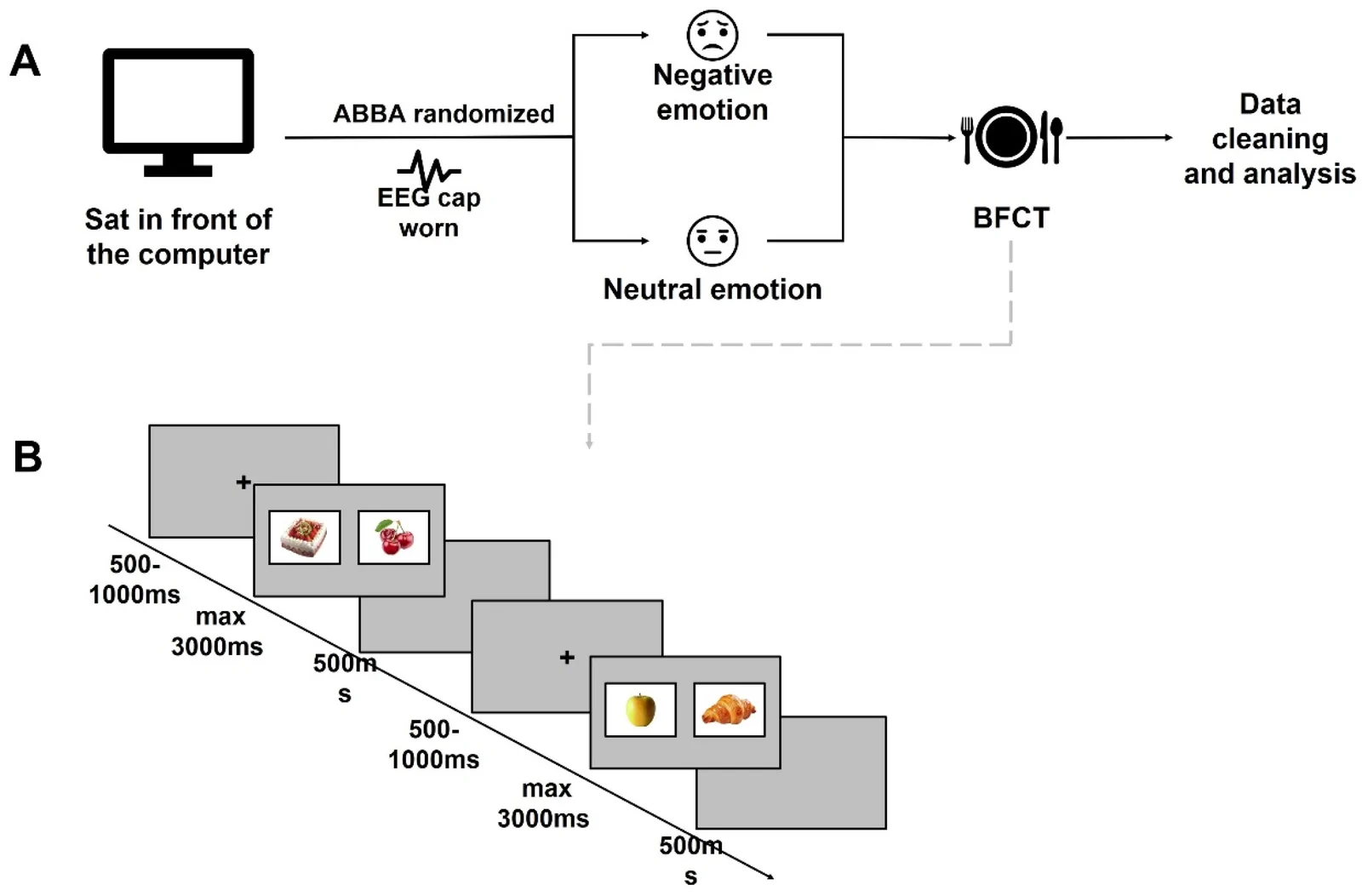
The experimental flow chart. (**A**) Participants were randomly assigned using the ABBA method to complete the binary food choice task under different emotional conditions. (**B**) A flowchart of the binary food choice task procedure. BFCT = binary food choice task.

The BFCT was designed to assess participants’ choices between high-calorie and low-calorie foods under deliciousness-congruent and deliciousness-incongruent conditions (Zhang et al., 2019). In each trial, participants were presented with one high-calorie item and one low-calorie item. Trials included both congruent pairs, in which the two options were comparable in deliciousness, and incongruent pairs, in which the high-calorie option was more delicious than the low-calorie option. Participants were instructed to choose the food they most wanted to eat at that moment by pressing the “F” or “J” key. Each trial began with a central fixation cross presented for a random duration of 500–1000 ms, followed by the presentation of the food pair, which remained on the screen until a response was made or 3000 ms had elapsed. A 500 ms inter-stimulus interval followed each response (Figure 1B). In the formal BFCT, participants completed 180 trials in total, including 60 deliciousness-congruent trials and 120 deliciousness-incongruent trials. Trial order was randomized for each participant, and the left/right positions of the high-calorie and low-calorie foods were counterbalanced across trials to reduce possible response-side bias.

### 2.4. Procedure

Participants were instructed to fast for approximately four hours prior to the experimental session, during which they were not allowed to consume anything except water. Upon arrival at the laboratory, the experimenter asked participants whether they had complied with the fasting instruction. Experimental sessions were scheduled according to participants’ appointments. Participants were seated in a well-lit, sound-attenuated room at a viewing distance of 100 cm from the monitor, and the experiment was presented using E-Prime 3.

The experimental paradigm consisted of two primary phases: emotion induction and a subsequent food-related decision task (Figure 1). To minimize potential order effects, a counterbalanced ABBA design was implemented, in which participants were randomly assigned to complete the negative and neutral emotional blocks in different orders. The same negative and neutral videos were presented to all participants. Between emotional blocks, participants were given a self-paced rest interval and were instructed to start the next block only when they felt ready to proceed. Participants completed the PANAS and VAS ratings before and after each emotion induction block to assess their affective state, hunger, thirst, and desire to eat. Prior to the formal experiment, participants completed a standardized practice session to ensure that they understood the task requirements and response procedure.

### 2.5. EEG Recording and Analyses

Scalp electroencephalographic (EEG) activity was continuously recorded using a 64-channel active electrode system (Brain Products, Munich, Germany). Electrodes were positioned according to the international 10–20 system. EEG data were recorded at a sampling rate of 1000 Hz, and electrode impedances were maintained below 5 kΩ throughout the recording. EEG data were processed in MATLAB (The MathWorks, Inc., Natick, MA, USA) using the EEGLAB and ERPLAB toolboxes (Delorme & Makeig, 2004; Lopez-Calderon & Luck, 2014). The data were re-referenced to the average of the bilateral mastoids and band-pass filtered from 0.1 to 45 Hz. Independent component analysis (ICA) was performed using the runica algorithm implemented in EEGLAB, and components contaminated by electrooculographic (EOG) artifacts, such as eye movements and blinks, as well as head movement artifacts, were removed from further analysis.

Stimulus-locked epochs were extracted from 200 ms before to 1000 ms after stimulus onset, with the 200 ms pre-stimulus interval used for baseline correction. After visual inspection, epochs with large amplitude fluctuations or voltage fluctuations exceeding ±100 μV at any electrode were rejected. No participant was excluded because of excessive EEG artifacts or insufficient valid trials. No systematic bad-channel problem was observed during preprocessing. The average numbers of accepted trials were 41.10 (*SD* = 9.91), 86.62 (*SD* = 17.23), 41.74 (*SD* = 8.19), and 85.24 (*SD* = 14.56) for the neutral-congruent, neutral-incongruent, negative-congruent, and negative-incongruent conditions, respectively. Only epochs with valid behavioral responses were included in ERP averaging.

ERP analyses were conducted on mean amplitudes extracted from the predefined parietal midline electrode site Pz. This site was selected because the present study focused on stimulus-locked parietal ERP activity during food decision-making, particularly the P3 component, which is typically maximal over centro-parietal or parietal midline regions (Hruby & Marsalek, 2003; Polich, 2007). Mean amplitudes of the N1, N2, and P3 components were extracted at Pz within their respective time windows: N1, 100–130 ms; N2, 170–220 ms; and P3, 280–380 ms.

### 2.6. Data Analysis

Prior to the main statistical analyses, the distributions of the dependent variables were examined using Shapiro–Wilk tests and Q–Q plots. Although some variables showed statistically significant deviations from normality, visual inspection of the Q–Q plots did not indicate severe departures from normality. Given the relatively balanced design and the robustness of ANOVA F tests to moderate deviations from normality, the planned ANOVA approach was retained (Blanca et al., 2017).

All statistical analyses were conducted using IBM SPSS Statistics 27.0 (IBM Corp., Armonk, NY, USA) First, independent-samples *t* tests were conducted on BMI and WHR to confirm the validity of group classification. To assess the effectiveness of emotion induction, PANAS scores and VAS happiness and sadness ratings were analyzed using 2 (time: pre- vs. post-induction) × 2 (emotion induction condition: negative vs. neutral) repeated-measures ANOVAs. Because hunger, thirst, and desire to eat were control variables related to eating motivation, they were analyzed separately using 2 (group: OWs vs. NWs) × 2 (time: before and after) × 2 (emotion: neutral and negative emotion) mixed-design ANOVAs.

Behavioral and ERP data were analyzed using 2 (group: OWs vs. NWs) × 2 (emotion: negative vs. neutral) × 2 (congruency: deliciousness-congruent vs. deliciousness-incongruent) ANOVAs. For the behavioral data, the dependent variables were the proportion of high-calorie choices and reaction times for high-calorie choice trials. For the ERP data, the dependent variables were the mean amplitudes of the N1, N2, and P3 components calculated within predefined ROI and time windows. Type III sums of squares were used for all analyses, and Greenhouse–Geisser corrections were applied when the assumption of sphericity was violated. Partial eta squared (*η*^2^*_p_*) was reported as the measure of effect size. Significant main effects and interactions were further examined, where appropriate, using simple main-effects analyses and comparisons adjusted with the Bonferroni correction.

## 3. Results

### 3.1. Self-Report Results

Independent-samples *t* test results showed that BMI was significantly higher in the OW group than in the NW group (OWs: *M* = 26.82, *SD* = 2.39; NWs: *M* = 20.36, *SD* = 1.49; *t*(37.90) = 11.35, *p* < 0.001). WHR was also significantly higher in the OW group than in the NW group (OWs: *M* = 0.79, *SD* = 0.05; NWs: *M* = 0.76, *SD* = 0.05; *t*(48) = 2.31, *p* = 0.025).

**Figure 2:**
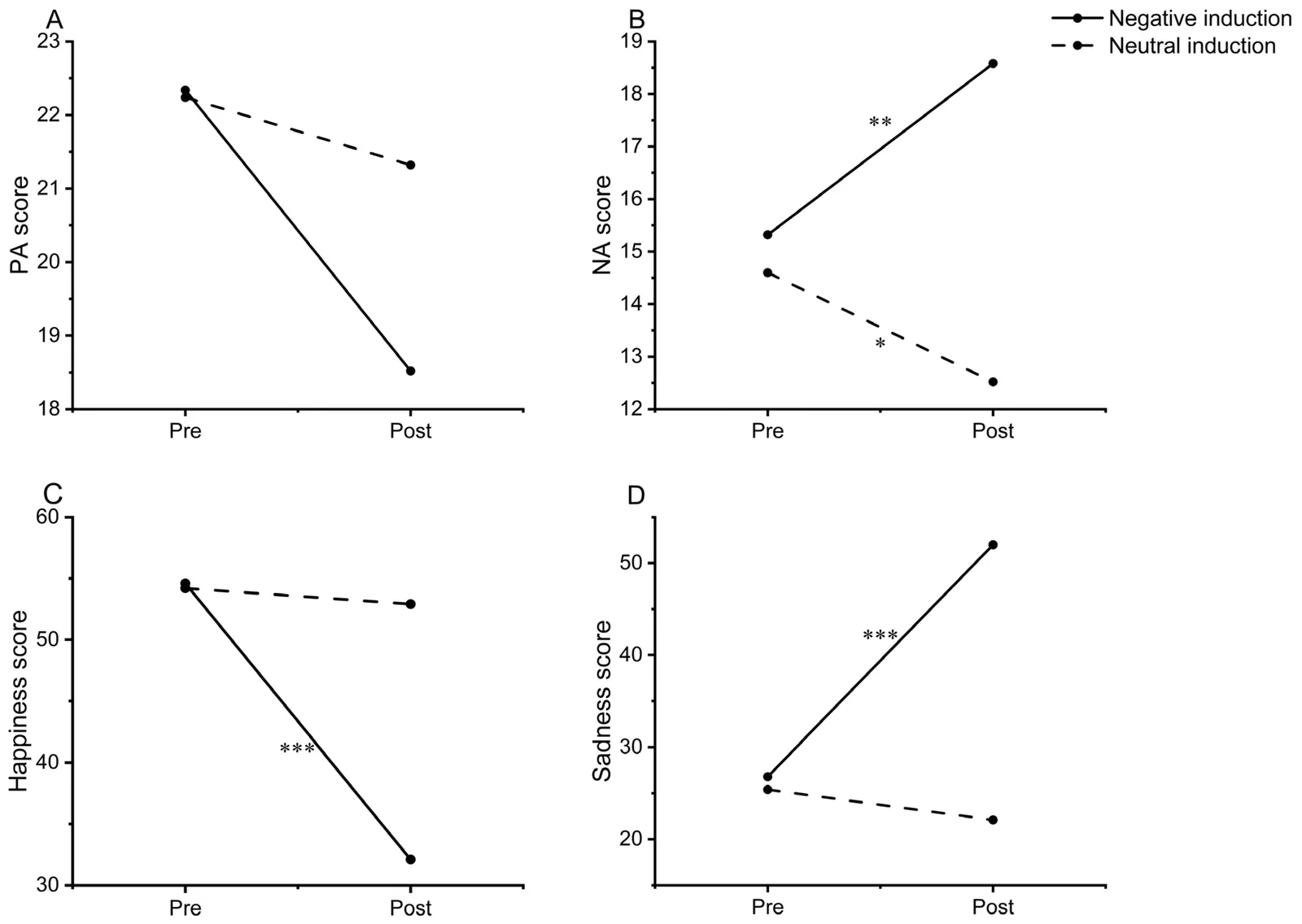
Changes in self-reported emotional states before and after emotion induction. (**A**) PA scores, (**B**) NA scores, (**C**) happiness scores, and (**D**) sadness scores before and after negative and neutral emotion induction. PA = positive affect; NA = negative affect. Pre = before emotion induction; Post = after emotion induction. * *p* < 0.05, ** *p* < 0.01, and *** *p* < 0.001.

Repeated-measures ANOVA showed a significant main effect of time on PA scores (*F*(1, 98) = 6.07, *p* = 0.015, *η*^2^*_p_* = 0.06, 95% CI [0.00, 0.17]), indicating that PA scores significantly decreased after emotion induction (Figure 2A).

For NA scores, there was a significant interaction between time and emotion induction condition (*F*(1, 98) = 13.84, *p* < 0.001, *η*^2^*_p_* = 0.12, 95% CI [0.03, 0.25]). Simple-effects analyses showed that NA scores significantly increased following negative emotion induction (*F*(1, 98) = 10.32, *p* = 0.002, *η*^2^*_p_* = 0.10, 95% CI [0.01, 0.21]), whereas NA scores significantly decreased following neutral emotion induction (*F*(1, 98) = 4.20, *p* = 0.043, *η*^2^*_p_* = 0.04, 95% CI [0.00, 0.14]). Further comparisons showed that, after emotion induction, NA scores were significantly higher in the negative emotion induction condition than in the neutral emotion induction condition (*F*(1, 98) = 38.87, *p* < 0.001, *η*^2^*_p_* = 0.28, 95% CI [0.14, 0.41]; Figure 2B).

For happiness scores, there was a significant interaction between time and emotion induction condition (*F*(1, 98) = 12.98, *p* < 0.001, *η*^2^*_p_* = 0.12, 95% CI [0.02, 0.24]). Simple-effects analyses showed that happiness scores significantly decreased after negative emotion induction (*F*(1, 98) = 29.23, *p* < 0.001, *η*^2^*_p_* = 0.23, 95% CI [0.10, 0.36]), but did not change significantly after neutral emotion induction (*F*(1, 98) = 0.10, *p* = 0.755, *η*^2^*_p_* = 0.00, 95% CI [0.00, 0.05]). Further comparisons showed that, after emotion induction, happiness scores were significantly lower in the negative emotion induction condition than in the neutral emotion induction condition (*F*(1, 98) = 27.35, *p* < 0.001, *η*^2^*_p_* = 0.22, 95% CI [0.09, 0.35]; Figure 2C).

For sadness scores, there was a significant interaction between time and emotion induction condition (*F*(1, 98) = 20.06, *p* < 0.001, *η*^2^*_p_* = 0.17, 95% CI [0.05, 0.30]). Sadness scores significantly increased after negative emotion induction (*F*(1, 98) = 31.37, *p* < 0.001, *η*^2^*_p_* = 0.24, 95% CI [0.11, 0.37]). In addition, after emotion induction, sadness scores were significantly higher in the negative emotion induction condition than in the neutral emotion induction condition (*F*(1, 98) = 40.82, *p* < 0.001, *η*^2^*_p_* = 0.29, 95% CI [0.15, 0.42]; Figure 2D).

Repeated-measures ANOVAs with time as the within-subject factor and group as the between-subject factor showed no significant main effects of time or group, and no significant interaction between time and group for hunger, thirst, or desire to eat ratings (*ps* > 0.05). These results indicate that participants’ subjective eating motivation remained relatively stable throughout the experimental session and did not differ significantly between the OW and NW groups.

### 3.2. Behavioral Results

**Figure 3:**
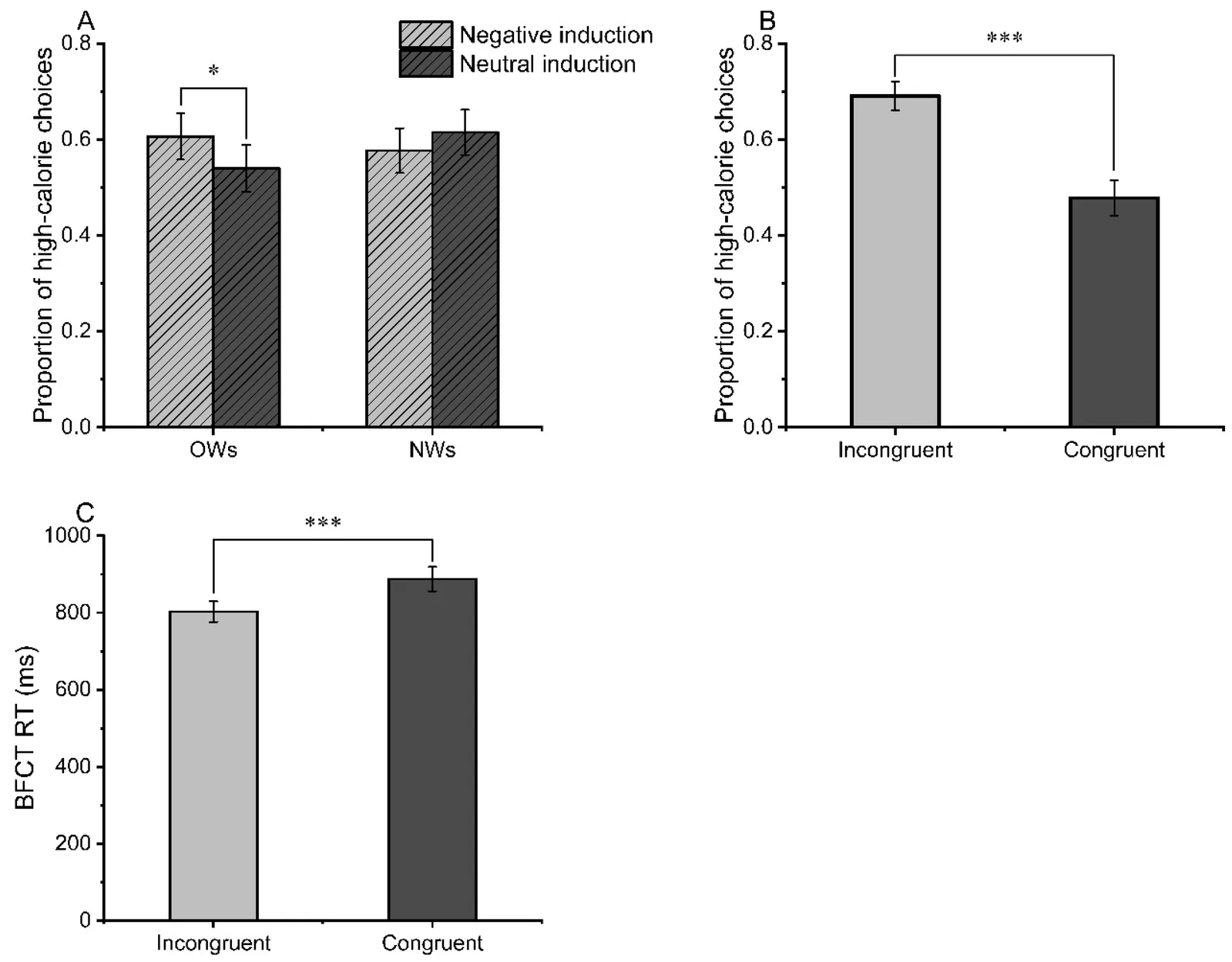
The proportion of high-calorie choices in the binary food choice task. (**A**) Main effect of deliciousness congruency. (**B**) The interaction between emotion and group. (**C**) Reaction times for high-calorie choice trials in the binary food choice task. OWs = overweight individuals; NWs = normal-weight individuals. BFCT = binary food choice task; RT = reaction time. Error bars represent standard errors of the mean. * *p* < 0.05; *** *p* < 0.001.

For the proportion of high-calorie food choices, the ANOVA revealed a significant interaction between emotion and group (*F*(1, 48) = 5.39, *p* = 0.025, *η*^2^*_p_* = 0.10, 95% CI [0.00, 0.27]). Simple-effects analyses showed that participants in the OW group chose high-calorie foods more frequently in the negative emotion condition than in the neutral emotion condition (*F*(1, 48) = 4.19, *p* = 0.046, *η*^2^*_p_* = 0.08, 95% CI [0.00, 0.25]), whereas no significant difference between emotion conditions was observed in the NW group (Figure 3A). There was a significant main effect of congruency (*F*(1, 48) = 97.29, *p* < 0.001, *η*^2^*_p_* = 0.67, 95% CI [0.50, 0.76]). Participants chose high-calorie foods more frequently in the deliciousness-incongruent condition than in the deliciousness-congruent condition (Figure 3B).

For reaction times on high-calorie choice trials in the BFCT, the results revealed a significant main effect of congruency (*F*(1, 48) = 22.23, *p* < 0.001, *η*^2^*_p_* = 0.32, 95% CI [0.11, 0.49]). Reaction times were significantly faster in the deliciousness-incongruent condition than in the deliciousness-congruent condition (Figure 3C).

### 3.3. ERP Results

#### 3.3.1. N1

The ANOVA results showed that the main effect of deliciousness congruency was significant for the N1 mean amplitudes (*F*(1, 48) = 7.46, *p* = 0.009, *η*^2^*_p_* = 0.13, 95% CI [0.01, 0.31]). The N1 mean amplitudes were significantly less negative in the deliciousness-incongruent condition.

#### 3.3.2. N2

In terms of the N2 mean amplitudes, no significant effects were observed, except for a marginally significant main effect of deliciousness congruency (*F*(1, 48) = 3.18, *p* = 0.081, *η*^2^*_p_* = 0.06, 95% CI [0.00, 0.22]). The N2 mean amplitudes were less negative in the deliciousness-incongruent condition.

#### 3.3.3. P3

In terms of the P3 mean amplitudes, the interaction between emotion and group was significant (*F*(1, 48) = 6.01, *p* = 0.018, *η*^2^*_p_* = 0.11, 95% CI [0.00, 0.28]). Simple-effects analyses showed that, in the NW group, the P3 mean amplitudes were significantly less positive under the negative emotion condition (*F*(1, 48) = 15.42, *p* < 0.001, *η*^2^*_p_* = 0.24, 95% CI [0.06, 0.42]). No significant difference was observed in the OW group.

**Figure 4:**
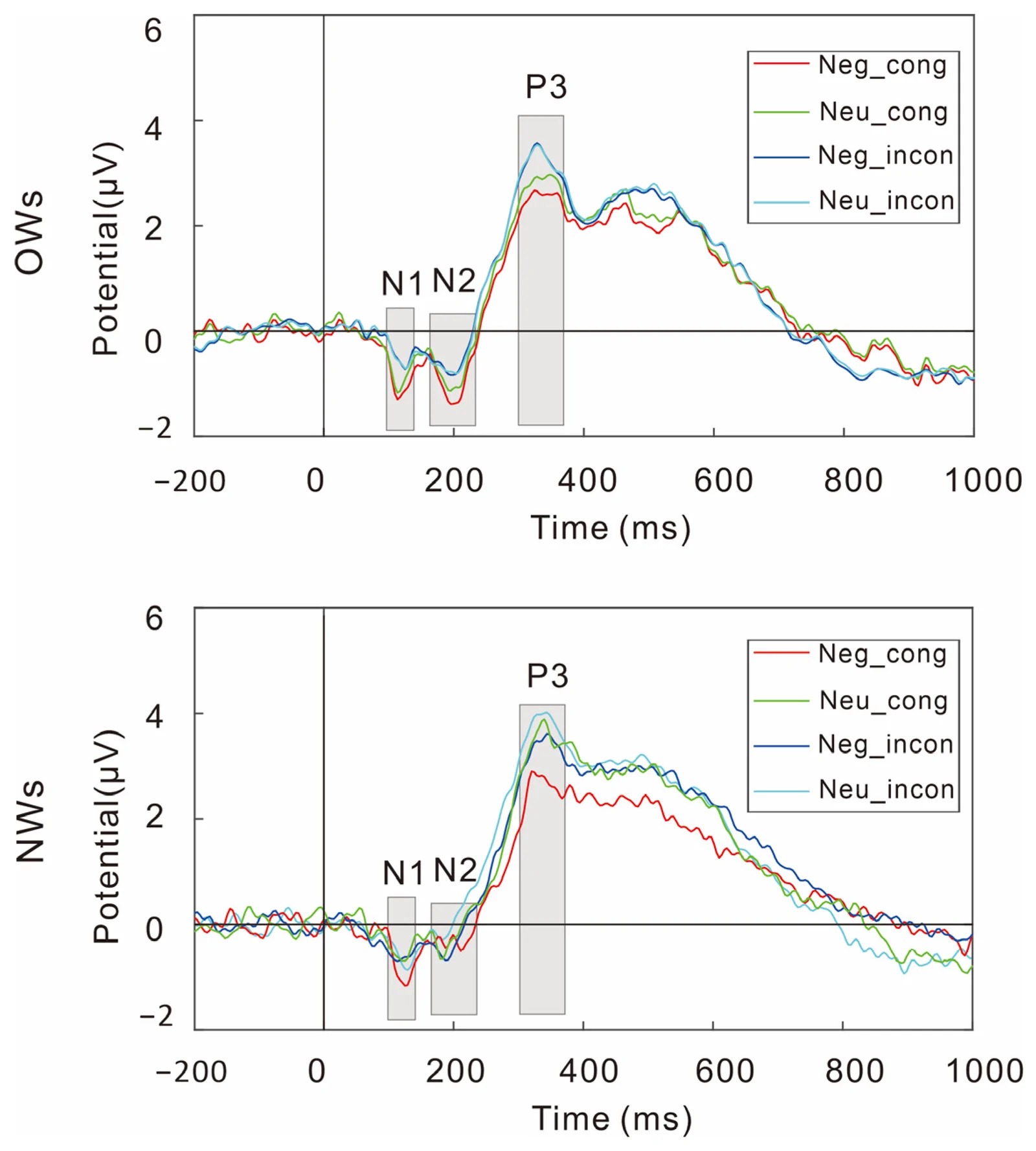
ERP waveforms at Pz for the OWs and NWs groups. OWs = overweight individuals; NWs = normal-weight individuals; Neg = negative emotion; Neu = neutral emotion; cong = congruent condition; incon = incongruent condition. Shaded areas indicate the N1, N2, and P3 time windows: 100–130 ms, 170–220 ms, and 280–380 ms, respectively.

The interaction between emotion and congruency was significant for P3 mean amplitudes (*F*(1, 48) = 5.72, *p* = 0.021, *η*^2^*_p_* = 0.11, 95% CI [0.00, 0.28]). Simple-effects analyses showed that, under both congruency conditions, P3 mean amplitudes were significantly less positive in the negative emotion condition than in the neutral emotion condition (congruent condition: *F*(1, 48) = 12.26, *p* = 0.001, *η*^2^*_p_* = 0.20, 95% CI [0.04, 0.38]; incongruent condition: *F*(1, 48) = 4.53, *p* = 0.038, *η*^2^*_p_* = 0.09, 95% CI [0.00, 0.25]). Further comparisons showed that only in the negative emotion condition, the deliciousness-incongruent condition elicited significantly more positive P3 mean amplitudes than the deliciousness-congruent condition (*F*(1, 48) = 19.19, *p* < 0.001, *η*^2^*_p_* = 0.29, 95% CI [0.09, 0.46]). No significant difference between congruency conditions was observed under neutral emotion. The ERP waveforms at Pz are shown in Figure 4.

## 4. Discussion

The present study examined how negative emotion and food deliciousness congruency influence high-calorie food choices among overweight and normal-weight female college students, as well as the neural mechanisms underlying the cognitive processes involved in food decision-making. By using an emotion induction paradigm and the BFCT, the present study yielded several main findings. First, deliciousness congruency significantly influenced food choice. When high-calorie foods were more delicious than low-calorie foods, participants chose high-calorie foods more frequently and responded faster. Second, negative emotion increased high-calorie food choices only among OWs, whereas this effect was not observed among NWs. Finally, the ERP results showed that the deliciousness-incongruent condition elicited less negative N1 and N2 amplitudes, although the effect for N2 was only marginally significant. P3 amplitudes were modulated by emotion, group, and deliciousness congruency, suggesting that late evaluative processing during food decision-making is sensitive to both affective state and individual weight status. Taken together, high-calorie food choices are influenced not only by calorie content, but also by the interaction between emotional state and deliciousness, particularly among OWs.

The behavioral results of the present study indicate that food deliciousness plays an important role in decisions involving high-calorie foods. From a reward learning perspective, this behavioral pattern may reflect the learned incentive value of palatable high-calorie foods. Repeated experiences with palatable foods may strengthen associations between food cues and rewarding eating outcomes, so that foods with higher perceived deliciousness become more likely to elicit eating desire and approach tendencies. Previous studies support this view. Using a virtual food shopping task, Mergelsberg et al. (2018) found that female college students chose delicious foods more quickly after categorizing foods according to deliciousness, and that this effect was independent of food healthiness, a finding consistent with the present results. In addition, Turnwald and Crum (2019) found that, compared with labels emphasizing health attributes, labels emphasizing taste, satisfaction, and pleasurable experience were more effective in increasing the selection of healthy foods and improving individuals’ evaluations of how delicious healthy foods were. According to the goal conflict model of eating, deliciousness may be a more salient driving factor in the goal conflict between enjoying palatable foods and controlling body weight. In other words, the advantage of high-calorie foods does not arise solely from their caloric properties; rather, it is further strengthened when they also have a deliciousness advantage.

The present study further found that the effect of negative emotion on high-calorie food choices differed by weight status. For OWs, negative emotion may be more likely to trigger an eating tendency in which food reward is used as a means of emotion regulation. One possible hypothesis is that this process may reduce the relative influence of weight control goals and bias food decision-making toward high-calorie foods (Vasileiou & Abbott, 2023). Another possible explanation is that NWs may have had healthier prior eating experiences and stronger cognitive control when facing the incentive value associated with high-calorie foods, which may have remained stronger than that of OWs even under negative emotion. This finding suggests that negative emotion may not universally increase preferences for high-calorie foods across all individuals, and its effect appeared to be more evident among OWs in the present study.

However, because potentially relevant variables such as emotional eating tendencies, dietary restraint, impulsivity, binge-eating symptoms, depressive symptoms, anxiety, were not measured, this group difference should not be attributed entirely to weight status. One possible explanation is that overweight status may be associated with other psychological characteristics, such as higher impulsivity under negative emotion, which could contribute to increased high-calorie food choices. Future studies should directly measure these variables to determine whether they mediate or moderate the association among weight status, negative emotion, food choice, and ERP responses.

The N1 component is associated with early visual perceptual processing and attentional selection. Less negative N1 amplitudes may suggest that when high-calorie foods have greater deliciousness, early perceptual or attentional processing of the food options may be modulated by differences in subjective reward value. The N2 component is commonly associated with conflict monitoring and response inhibition. The trend toward less negative N2 amplitudes in the deliciousness-incongruent condition may suggest that, when high-calorie foods have a clear deliciousness advantage, the choice process may involve relatively reduced goal conflict and lower demands for conflict monitoring. However, because this effect was only marginally significant, this interpretation should be treated with caution. Consistent with this interpretation, Meule et al. (2013) and Y. Liu et al. (2022) found that high-calorie foods elicited less negative N1 and N2 amplitudes, respectively. These findings suggest that when foods possess features with greater subjective reward value, such as high calorie content or high deliciousness, individuals’ motivational tendency may shift more strongly toward enjoying palatable foods. Under such circumstances, the choice goal may become relatively clearer, and the demands on early attentional allocation and conflict monitoring may be lower; consequently, individuals may complete the choice with comparatively less cognitive effort. However, ERP responses often reflect complex neurocognitive processing rather than a single cognitive process, and therefore, our interpretations of the N1 and N2 findings should be regarded as tentative and treated with caution.

Regarding late ERP components, the present study found that P3 amplitudes were modulated by emotion, weight status, and deliciousness congruency. In the NW group, negative emotion significantly reduced P3 amplitudes. This pattern may suggest that negative emotion was associated with reduced cognitive resources available for food cue evaluation and decision-related processing. For NWs, such reduced late ERP responses may reflect decreased sensitivity to food cues or reduced evaluative processing, which may partly explain why negative emotion did not increase high-calorie food choices at the behavioral level. In contrast, OWs did not show a significant reduction in P3 amplitudes under negative emotion. One possible explanation is that they may have maintained relatively high sensitivity to food cues in a negative emotional state. Previous meta-analytic studies have consistently shown that individuals with obesity or overweight exhibit stronger responses in brain regions associated with reward processing when exposed to food-related stimuli, especially high-calorie food cues, whereas NWs show relatively greater responses in regions associated with inhibitory control (Devoto et al., 2018; Meng et al., 2020). In addition, the interaction between emotion and deliciousness congruency further suggests that late evaluative processing was also sensitive to the subjective reward value of food cues. Specifically, under negative emotion, high-calorie foods with a deliciousness advantage elicited larger P3 amplitudes, indicating that perceived deliciousness may continue to enhance late-stage food evaluation even when negative emotion reduces overall processing resources. Therefore, the absence of a P3 reduction among OWs under negative emotion may reflect their sustained attentional and evaluative processing of reward-related food cues, thereby contributing to a behavioral tendency to choose high-calorie foods. However, because reward sensitivity, evaluative processing, and weight control goals were not directly measured in the present study, these interpretations are based on theoretical inference and should be treated with caution. Future studies should directly measure these variables to further examine the proposed mechanisms.

Overall, the present study demonstrates, at both the behavioral and ERP levels, that negative emotion, deliciousness advantage, and weight status jointly influence high-calorie food decision-making. A deliciousness advantage increased high-calorie food choices and accelerated reaction times, whereas negative emotion primarily enhanced the tendency to choose high-calorie foods among OWs. The ERP results further indicate that a deliciousness advantage not only affects early attentional processing and conflict monitoring, but also modulates late evaluative processing under negative emotional states. Thus, high-calorie food decision-making is not determined solely by the caloric properties of foods, but is shaped by the combined influence of subjective reward value, emotional state, and weight-related characteristics. These findings provide insights into unhealthy eating behaviors associated with obesity. These findings suggest that future research and intervention development for OWs may benefit from considering negative emotion management and perceived food deliciousness. However, because the present study used a laboratory-based experimental design rather than a longitudinal or clinical intervention design, these practical implications should be regarded as preliminary and require further validation in future intervention studies.

Several limitations of the present study should be acknowledged. First, the study recruited only female college students, resulting in a relatively homogeneous sample in terms of demographic characteristics. In addition, the sample size was relatively small for ERP research. Therefore, whether the findings can be generalized to other populations requires further validation. Future studies could expand the sample to include individuals of different genders, ages, and weight statuses, thereby examining the generalizability and individual differences in the effects of emotion and deliciousness congruency on food decision-making. Second, the present study classified participants into overweight and normal-weight groups primarily based on BMI. Although WHR was calculated and reported as an additional anthropometric indicator, other adiposity-related measures, such as body fat percentage or waist-to-height ratio, were not assessed. Therefore, the interpretation of weight-status-related effects should be treated with caution. Third, the ecological validity of the laboratory-based food choice paradigm is limited. The present study used a food-picture choice task to examine preferences for high-calorie foods. Although this task allows for relatively good control over food attributes and enables the recording of the decision-making process, choices based on food pictures are not equivalent to actual eating behavior. Future research could incorporate actual food intake, food purchasing behavior, or ecological momentary assessment methods to further improve ecological validity. Fourth, laboratory-based emotion induction may not fully capture the effects of more complex negative emotional experiences in daily life, such as chronic stress, anxiety, or depression, on eating behavior. Future studies could further distinguish between different types and durations of negative emotion and examine their differential effects on high-calorie food choices among OWs. Fifth, the present study involved multiple behavioral, self-report, and ERP analyses, which may increase the possibility of false positive findings due to multiple testing. Therefore, the results should be interpreted with caution and further replicated in future studies. Finally, the present study was not preregistered, and the data are not publicly available due to privacy and ethical restrictions, which may limit transparency and independent reproducibility.

## 5. Conclusions

The present study showed that high-calorie food decision-making varied as a function of negative emotion, deliciousness congruency, and weight status. At the behavioral level, when high-calorie foods were more delicious than low-calorie foods, individuals were more likely to choose high-calorie foods and responded more quickly. Meanwhile, OWs showed a higher tendency to choose high-calorie foods under negative emotion compared to under neutral emotion. At the ERP level, a deliciousness advantage was associated with less negative N1 amplitudes and marginally less negative N2 amplitudes, which may reflect differences in early attentional and conflict-related processing. The P3 results further suggest that emotional state, deliciousness congruency, and weight status were associated with differences in late ERP responses, which may be related to evaluative processing during food decision-making. Overall, high-calorie food choices are related not only to caloric properties, but also to the subjective reward value of foods and individuals’ emotional states. For OWs, negative emotion may be associated with an increased bias toward high-calorie food choices. In addition, high-calorie foods with a deliciousness advantage are generally more likely to be selected. The present study provides ERP evidence regarding the temporal correlates of high-calorie food decision-making under negative emotion. These findings may inform future research on interventions targeting unhealthy eating behaviors, particularly those considering emotional states and perceived food deliciousness.

## Data Availability

The data presented in this study are available on request from the corresponding author. The data are not publicly available due to privacy and ethical restrictions.

## Abbreviations

The following abbreviations are used in this manuscript:

BFCT	Binary food choice task
BMI	Body mass index
EEG	Electroencephalography
ERP	Event-related potential
NA	Negative affect
NWs	Normal-weight individuals
OWs	Overweight individuals
PA	Positive affect
PANAS	Positive and Negative Affect Schedule
ROI	Region of interest
VAS	Visual analog scale
WHO	World Health Organization

## References

[B1-behavsci-16-01265] Bian Z., Yang R., Yang X., Liu Y., Gao X., Chen H. (2021). Influence of negative mood on restrained eaters’ memory suppression of food cues: An event-related potentials study. Appetite.

[B2-behavsci-16-01265] Bianco V., Veniero D., D’Acunto A., Koch G., Picazio S. (2023). Challenging inhibitory control with high- and low-calorie food: A behavioural and TMS study. Frontiers in Nutrition.

[B3-behavsci-16-01265] Biehl S. C., Keil J., Naumann E., Svaldi J. (2020). ERP and oscillatory differences in overweight/obese and normal-weight adolescents in response to food stimuli. Journal of Eating Disorders.

[B4-behavsci-16-01265] Blanca M. J., Alarcón R., Arnau J., Bono R., Bendayan R. (2017). Non-normal data: Is ANOVA still a valid option?. Psicothema.

[B5-behavsci-16-01265] Blüher M. (2025). An overview of obesity-related complications: The epidemiological evidence linking body weight and comorbidities. Diabetes, Obesity and Metabolism.

[B6-behavsci-16-01265] Carbine K. A., Rodeback R., Modersitzki E., Miner M., LeCheminant J. D., Larson M. J. (2018). The utility of event-related potentials (ERPs) in understanding food-related cognition: A systematic review and recommendations. Appetite.

[B7-behavsci-16-01265] Chen P.-J., Antonelli M. (2020). Conceptual models of food choice: Influential factors related to foods, individual differences, and society. Foods.

[B8-behavsci-16-01265] Dakanalis A., Mentzelou M., Papadopoulou S. K., Papandreou D., Spanoudaki M., Vasios G. K., Pavlidou E., Mantzorou M., Giaginis C. (2023). The association of emotional eating with overweight/obesity, depression, anxiety/stress, and dietary patterns: A review of the current clinical evidence. Nutrients.

[B9-behavsci-16-01265] Delorme A., Makeig S. (2004). EEGLAB: An open source toolbox for analysis of single-trial EEG dynamics including independent component analysis. Journal of Neuroscience Methods.

[B10-behavsci-16-01265] Devoto F., Zapparoli L., Bonandrini R., Berlingeri M., Ferrulli A., Luzi L., Banfi G., Paulesu E. (2018). Hungry brains: A meta-analytical review of brain activation imaging studies on food perception and appetite in obese individuals. Neuroscience & Biobehavioral Reviews.

[B11-behavsci-16-01265] Fazzino T. L., Rohde K., Sullivan D. K. (2019). Hyper-palatable foods: Development of a quantitative definition and application to the US food system database. Obesity.

[B12-behavsci-16-01265] Fernqvist F., Spendrup S., Tellström R. (2024). Understanding food choice: A systematic review of reviews. Heliyon.

[B13-behavsci-16-01265] Flint A., Raben A., Blundell J., Astrup A. (2000). Reproducibility, power and validity of visual analogue scales in assessment of appetite sensations in single test meal studies. International Journal of Obesity.

[B14-behavsci-16-01265] Folstein J. R., Van Petten C. (2008). Influence of cognitive control and mismatch on the N2 component of the ERP: A review. Psychophysiology.

[B15-behavsci-16-01265] Fuente González C. E., Chávez-Servín J. L., de la Torre-Carbot K., Ronquillo González D., Aguilera Barreiro M. L. Á., Ojeda Navarro L. R. (2022). Relationship between emotional eating, consumption of hyperpalatable energy-dense foods, and indicators of nutritional status: A systematic review. Journal of Obesity.

[B16-behavsci-16-01265] Georgii C., Schulte-Mecklenbeck M., Richard A., Van Dyck Z., Blechert J. (2019). The dynamics of self-control: Within-participant modeling of binary food choices and underlying decision processes as a function of restrained eating. Psychological Research.

[B17-behavsci-16-01265] Ha O., Lim S. (2023). The role of emotion in eating behavior and decisions. Frontiers in Psychology.

[B18-behavsci-16-01265] Hare T. A., Camerer C. F., Rangel A. (2009). Self-control in decision-making involves modulation of the vmPFC valuation system. Science.

[B19-behavsci-16-01265] Hruby T., Marsalek P. (2003). Event-related potentials—The P3 wave. Acta Neurobiologiae Experimentalis.

[B20-behavsci-16-01265] Huang L., Yang T., Li Z. (2003). Applicability of the positive and negative affect scale in Chinese. Chinese Mental Health Journal.

[B21-behavsci-16-01265] Jun D., Girard J. M., Martin C. K., Fazzino T. L. (2025). The role of hyper-palatable foods in energy intake measured using mobile food photography methodology. Eating Behaviors.

[B22-behavsci-16-01265] Kirsten H., Seib-Pfeifer L., Gibbons H. (2022). Effects of the calorie content of visual food stimuli and simulated situations on event-related frontal alpha asymmetry and event-related potentials in the context of food choices. Appetite.

[B23-behavsci-16-01265] Klatzkin R. R., Nolan L. J., Kissileff H. R. (2021). Self-reported emotional eaters consume more food under stress if they experience heightened stress reactivity and emotional relief from stress upon eating. Physiology & Behavior.

[B24-behavsci-16-01265] Lerner J. S., Li Y., Valdesolo P., Kassam K. S. (2015). Emotion and decision making. Annual Review of Psychology.

[B25-behavsci-16-01265] Li X., Pan Y., Han Y., Liang Q., Yang X., Meng X., Gao X. (2022). Chinese food image database for eating and appetite studies. Nutrients.

[B26-behavsci-16-01265] Liu X., Liu Y., Shi H., Li L., Zheng M. (2021). Regulation of mindfulness-based music listening on negative emotions related to COVID-19: An ERP study. International Journal of Environmental Research and Public Health.

[B27-behavsci-16-01265] Liu Y., Roefs A., Nederkoorn C. (2021). Food palatability directs our eyes across contexts. Frontiers in Psychology.

[B28-behavsci-16-01265] Liu Y., Zhang L., Jackson T., Wang J., Yang R., Chen H. (2020). Effects of negative mood state on event-related potentials of restrained eating subgroups during an inhibitory control task. Behavioural Brain Research.

[B29-behavsci-16-01265] Liu Y., Zhao J., Zhou Y., Yang R., Han B., Zhao Y., Pang Y., Yuan H., Chen H. (2022). High-calorie food-cues impair conflict control: EEG evidence from a food-related stroop task. Nutrients.

[B30-behavsci-16-01265] Lopez-Calderon J., Luck S. J. (2014). ERPLAB: An open-source toolbox for the analysis of event-related potentials. Frontiers in Human Neuroscience.

[B31-behavsci-16-01265] Macht M. (2008). How emotions affect eating: A five-way model. Appetite.

[B32-behavsci-16-01265] Meng X., Huang D., Ao H., Wang X., Gao X. (2020). Food cue recruits increased reward processing and decreased inhibitory control processing in the obese/overweight: An activation likelihood estimation meta-analysis of fMRI studies. Obesity Research & Clinical Practice.

[B33-behavsci-16-01265] Mergelsberg E. L., MacLeod C., Mullan B., Rudaizky D., Allom V., Houben K., Lipp O. V. (2018). Food healthiness versus tastiness: Contrasting their impact on more and less successful healthy shoppers within a virtual food shopping task. Appetite.

[B34-behavsci-16-01265] Meule A., Kübler A., Blechert J. (2013). Time course of electrocortical food-cue responses during cognitive regulation of craving. Frontiers in Psychology.

[B35-behavsci-16-01265] Özbay A., Demirkol M. E., Tamam L., Namlı Z., Karaytuğ M. O., Yeşiloğlu C. (2025). The relationship between binge eating behavior and psychological pain in patients with major depressive disorder. Behavioral Sciences.

[B36-behavsci-16-01265] Paakki M., Kantola M., Junkkari T., Arjanne L., Luomala H., Hopia A. (2022). “Unhealthy = Tasty”: How does it affect consumers’ (un)healthy food expectations?. Foods.

[B37-behavsci-16-01265] Phelps N. H., Singleton R. K., Zhou B., Heap R. A., Mishra A., Bennett J. E., Paciorek C. J., Lhoste V. P., Carrillo-Larco R. M., Stevens G. A., Rodriguez-Martinez A., Bixby H., Bentham J., Di Cesare M., Danaei G., Rayner A. W., Barradas-Pires A., Cowan M. J., Savin S., Alshangiti A. M. (2024). Worldwide trends in underweight and obesity from 1990 to 2022: A pooled analysis of 3663 population-representative studies with 222 million children, adolescents, and adults. The Lancet.

[B38-behavsci-16-01265] Polich J. (2007). Updating P300: An integrative theory of P3a and P3b. Clinical Neurophysiology.

[B39-behavsci-16-01265] Raghunathan R., Naylor R. W., Hoyer W. D. (2006). The unhealthy = tasty intuition and its effects on taste inferences, enjoyment, and choice of food products. Journal of Marketing.

[B40-behavsci-16-01265] Reichenberger J., Schnepper R., Arend A. K., Blechert J. (2020). Emotional eating in healthy individuals and patients with an eating disorder: Evidence from psychometric, experimental and naturalistic studies. The Proceedings of the Nutrition Society.

[B41-behavsci-16-01265] Rouhani M. H., Haghighatdoost F., Surkan P. J., Azadbakht L. (2016). Associations between dietary energy density and obesity: A systematic review and meta-analysis of observational studies. Nutrition.

[B42-behavsci-16-01265] Schindler S., Schettino A., Pourtois G. (2018). Electrophysiological correlates of the interplay between low-level visual features and emotional content during word reading. Scientific Reports.

[B43-behavsci-16-01265] Schnepper R., Georgii C., Eichin K., Arend A.-K., Wilhelm F. H., Vögele C., Lutz A. P. C., van Dyck Z., Blechert J. (2020). Fight, flight,–or grab a bite! Trait emotional and restrained eating style predicts food cue responding under negative emotions. Frontiers in Behavioral Neuroscience.

[B44-behavsci-16-01265] Stroebe W. (2022). The goal conflict model: A theory of the hedonic regulation of eating behavior. Current Opinion in Behavioral Sciences.

[B45-behavsci-16-01265] Stroebe W., van Koningsbruggen G. M., Papies E. K., Aarts H. (2013). Why most dieters fail but some succeed: A goal conflict model of eating behavior. Psychological Review.

[B46-behavsci-16-01265] Sullivan N., Hutcherson C., Harris A., Rangel A. (2015). Dietary self-control is related to the speed with which attributes of healthfulness and tastiness are processed. Psychological Science.

[B47-behavsci-16-01265] Suzuki S. (2022). Constructing value signals for food rewards: Determinants and the integration. Current Opinion in Behavioral Sciences.

[B48-behavsci-16-01265] Turnwald B. P., Crum A. J. (2019). Smart food policy for healthy food labeling: Leading with taste, not healthiness, to shift consumption and enjoyment of healthy foods. Preventive Medicine.

[B49-behavsci-16-01265] van den Akker K., Schyns G., Jansen A. (2018). Learned overeating: Applying principles of Pavlovian conditioning to explain and treat overeating. Current Addiction Reports.

[B50-behavsci-16-01265] Van Der Laan L. N., De Ridder D. T. D., Charbonnier L., Viergever M. A., Smeets P. a. M. (2014). Sweet lies: Neural, visual, and behavioral measures reveal a lack of self-control conflict during food choice in weight-concerned women. Frontiers in Behavioral Neuroscience.

[B51-behavsci-16-01265] Vasileiou V., Abbott S. (2023). Emotional eating among adults with healthy weight, overweight and obesity: A systematic review and meta-analysis. Journal of Human Nutrition and Dietetics: The Official Journal of the British Dietetic Association.

[B52-behavsci-16-01265] Watson D., Clark L. A., Tellegen A. (1988). Development and validation of brief measures of positive and negative affect: The PANAS scales. Journal of Personality and Social Psychology.

[B53-behavsci-16-01265] Zhang X., Luo Y., Liu Y., Yang C., Chen H. (2019). Lack of conflict during food choice is associated with the failure of restrained eating. Eating Behaviors.

[B54-behavsci-16-01265] Zsoldos I., Sinding C., Chambaron S. (2022). Using event-related potentials to study food-related cognition: An overview of methods and perspectives for future research. Brain and Cognition.

